# Ascites as a Rare Manifestation of Malignant Peritoneal Mesothelioma: A Case Report

**DOI:** 10.7759/cureus.70982

**Published:** 2024-10-07

**Authors:** Sean Lief, Srihita Patibandla, Ali Z Ansari, Nilay Bhatt, Azouba Gulraiz, Samer M Beauti, Rashad Ali

**Affiliations:** 1 Department of Internal Medicine, William Carey University College of Osteopathic Medicine, Hattiesburg, USA; 2 Department of Internal Medicine, Trinity Health Grand Rapids, Grand Rapids, USA; 3 Department of Pathology, William Carey University College of Osteopathic Medicine, Hattiesburg, USA; 4 Department of Internal Medicine, HCA Houston Healthcare Clear Lake, Webster, USA; 5 Department of Internal Medicine, Poplar Bluff Regional Medical Center, Poplar Bluff, USA; 6 Department of Obstetrics and Gynecology, South Central Regional Medical Center, Laurel, USA

**Keywords:** abdominal paracentesis, asbestos exposure, ascites, atypical mesothelial cells, chronic abdominal pain, hepatic steatosis, lumbar spondylosis, malignant peritoneal mesothelioma, omental caking, phlebolith

## Abstract

Malignant peritoneal mesothelioma (MPM) is an aggressive neoplasm that originates from the mesothelial cells lining the parietal peritoneum or visceral peritoneum and extensively spreads within the abdominal cavity. It is a rare malignancy characterized by an insidious onset and poor prognosis. We present the case of a 79-year-old Caucasian male who experienced escalating abdominal pain for six weeks and acute abdominal distension. His medical history was significant for hypertension, gastroesophageal reflux disease (GERD), hypercholesterolemia, and prior coronary artery bypass grafting (CABG). The patient had a 30-pack-year smoking history and worked as a plumber and roofer until retirement. We also confirmed with the patient that he has never been diagnosed with asbestosis. He reported no family history of mesothelioma or related conditions. A computed tomography (CT) scan revealed a prior sternotomy, mild pleural calcifications, mild hepatic steatosis, diffuse peritoneal ascites, diffuse omental edema, and pelvic phleboliths. MPM was confirmed through histopathological examination, which revealed atypical mesothelial cells with high nucleus-to-cytoplasm ratios, prominent nucleoli, and irregular nuclear membranes. It also revealed tumor cells positive for p53, calretinin, WT1, and podoplanin (D2-40). This case highlights the importance of considering MPM in the differential diagnosis for patients with ascites and possible asbestos exposure, particularly with respect to occupational hazards, as it is a rare manifestation of the disease.

## Introduction

Mesothelioma is a malignancy that arises from pleural or peritoneal parietal or visceral membranes, which are the serous membranes lining various body cavities or organs respectively. While the commonly known pleural form of mesothelioma usually presents with chest pain and shortness of breath due to the tumor growth in the chest cavity, the much rarer peritoneal form of mesothelioma may present with ascites and abdominal distension. Malignant peritoneal mesothelioma (MPM) is a particularly rare and aggressive form of mesothelioma that affects the parietal peritoneum, the thin serous lining of the abdominal cavity. The annual incidence of MPM in the general population is approximately 1 case per 1 million people [1]. It predominantly affects males and is strongly linked to asbestos exposure, especially in occupational settings. Notably, symptoms often appear after a lengthy latency period, sometimes exceeding 40 years post-exposure. Despite advances in treatment, the prognosis for MPM remains poor, with a median survival rate of around one year [2].

While asbestos exposure is strongly associated with pleural mesothelioma, the link between asbestos and MPM is considered weaker. MPM accounts for only about 10% of all mesothelioma cases [2]. Diagnosing MPM requires a high degree of suspicion, as radiological and laboratory findings often lack specificity. The definitive diagnosis depends on histopathological examination, which identifies nodular tissue changes. Pathological diagnosis also provides insight into prognosis: the epithelioid type generally has a more favorable median survival of about 12 months, whereas the sarcomatoid type is associated with a median survival of approximately 5 months, the biphasic type carries a similar prognosis to the sarcomatoid type. The epithelioid subtype is characterized by cuboidal or columnar cells with a more organized structure, whereas the sarcomatoid subtype consists of spindle-shaped cells with a disorganized appearance. Additionally, lower levels of the Ki67 proliferating cellular antigen are linked to a better survival outlook [3]. Histopathology should include sensitive staining techniques using calretinin, WT1, and CK5/6 to enhance diagnostic accuracy. Computed tomography (CT) scans are the preferred imaging modality for detecting MPM [4]. Unfortunately, MPM often presents with no early clinical symptoms, leading to diagnosis at more advanced stages due to its unusual and aggressive progression.

## Case presentation

A 79-year-old Caucasian male presented to the emergency department with complaints of worsening abdominal pain. The patient reported that the pain had been ongoing for six weeks and had recently become intolerable. Initially, he rated the pain as 1/10 on the pain scale; however, it progressed to 8/10. The pain was non-radiating and did not respond to any form of relief. He also reported constant bloating, decreased appetite, generalized weakness, and early satiety. His medical history was notable for ascites, essential hypertension, gastroesophageal reflux disease (GERD), hypercholesterolemia, and prior coronary artery bypass grafting (CABG). The patient had a 30-pack-year smoking history but no current tobacco use. He reported no family history of mesothelioma or related conditions. On physical examination, he exhibited tenderness in all four quadrants of the abdomen and had a visibly distended abdomen. Despite the tenderness, there was no guarding or rebound tenderness observed. He also revealed that he had worked as a plumber and roofer from his 20s until retirement. We also confirmed with the patient that he has never been diagnosed with asbestosis. A fluid wave test was positive, indicating the presence of ascites.

**Figure 1:**
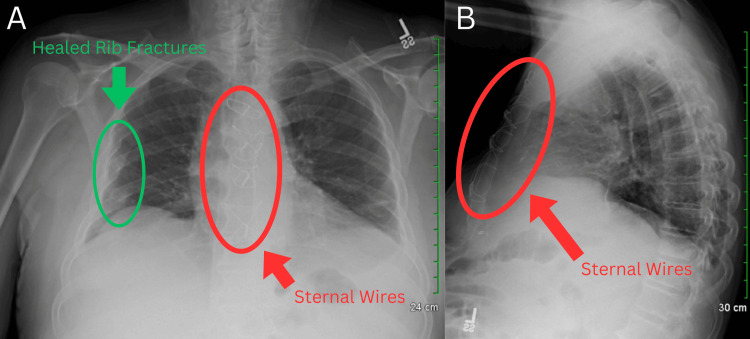
: Anteroposterior (A) and lateral (B) chest X-rays showing a normal cardiac silhouette, presence of sternal wires (A-B), old healed right rib fractures (A), reduced lung volumes, and clear lungs.

**Figure 2:**
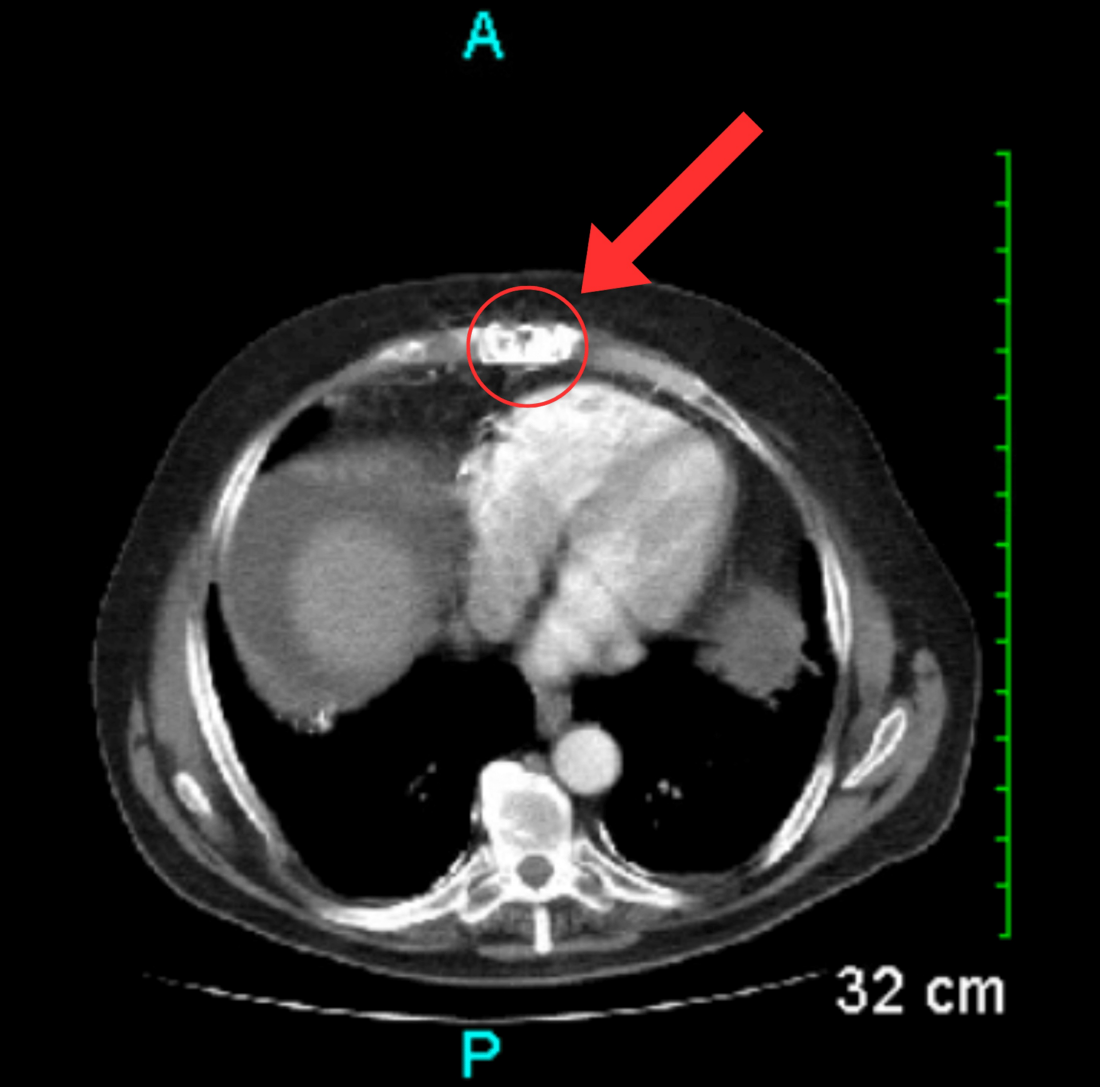
CT scan showing evidence of a prior sternotomy (red arrow and circle). CT: Computed tomography

**Figure 3:**
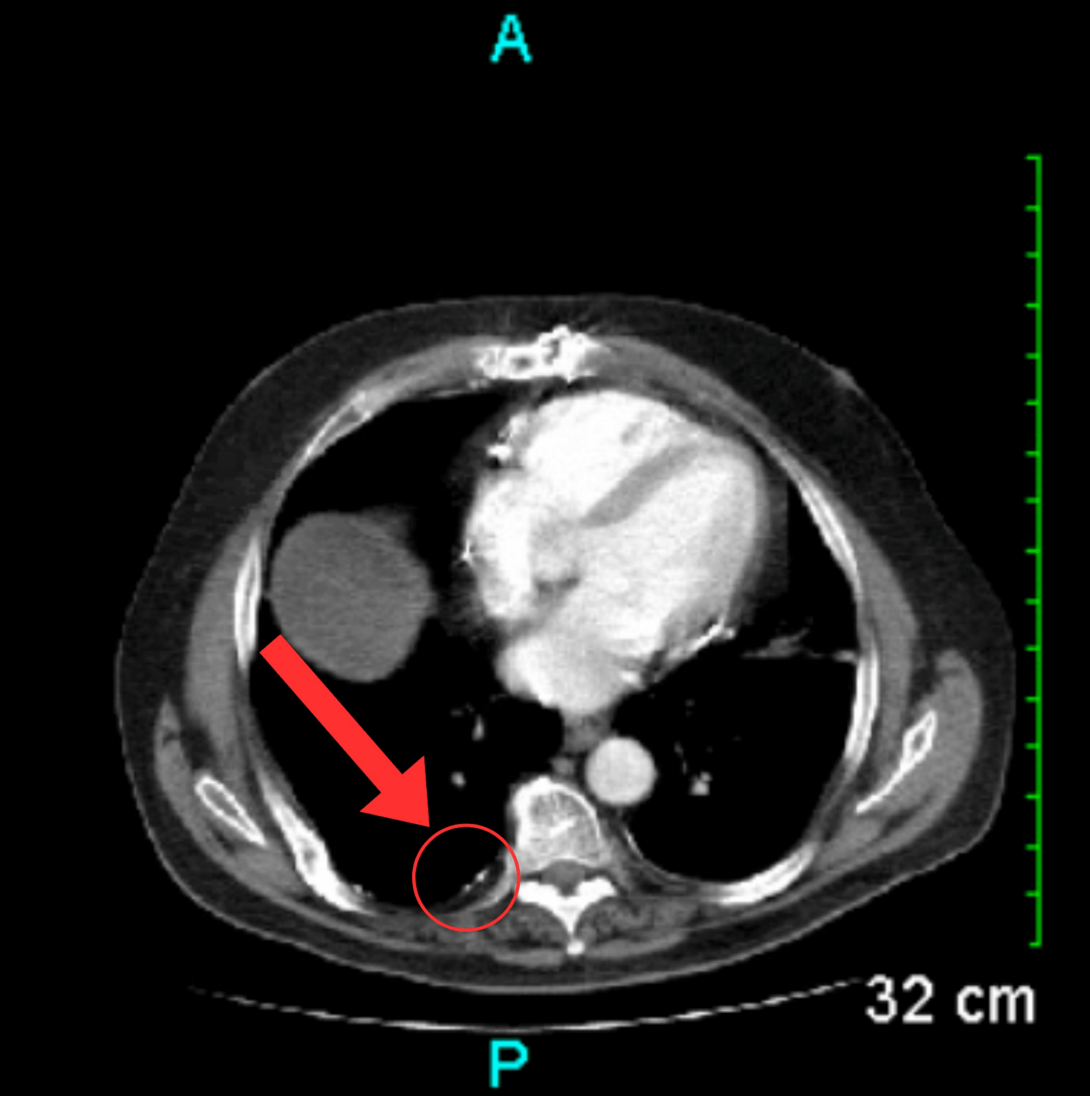
CT scan showing mild pleural calcifications posteriorly in the right hemithorax and over the posterior right hemidiaphragm (red arrow and circle). CT: Computed tomography

**Figure 4:**
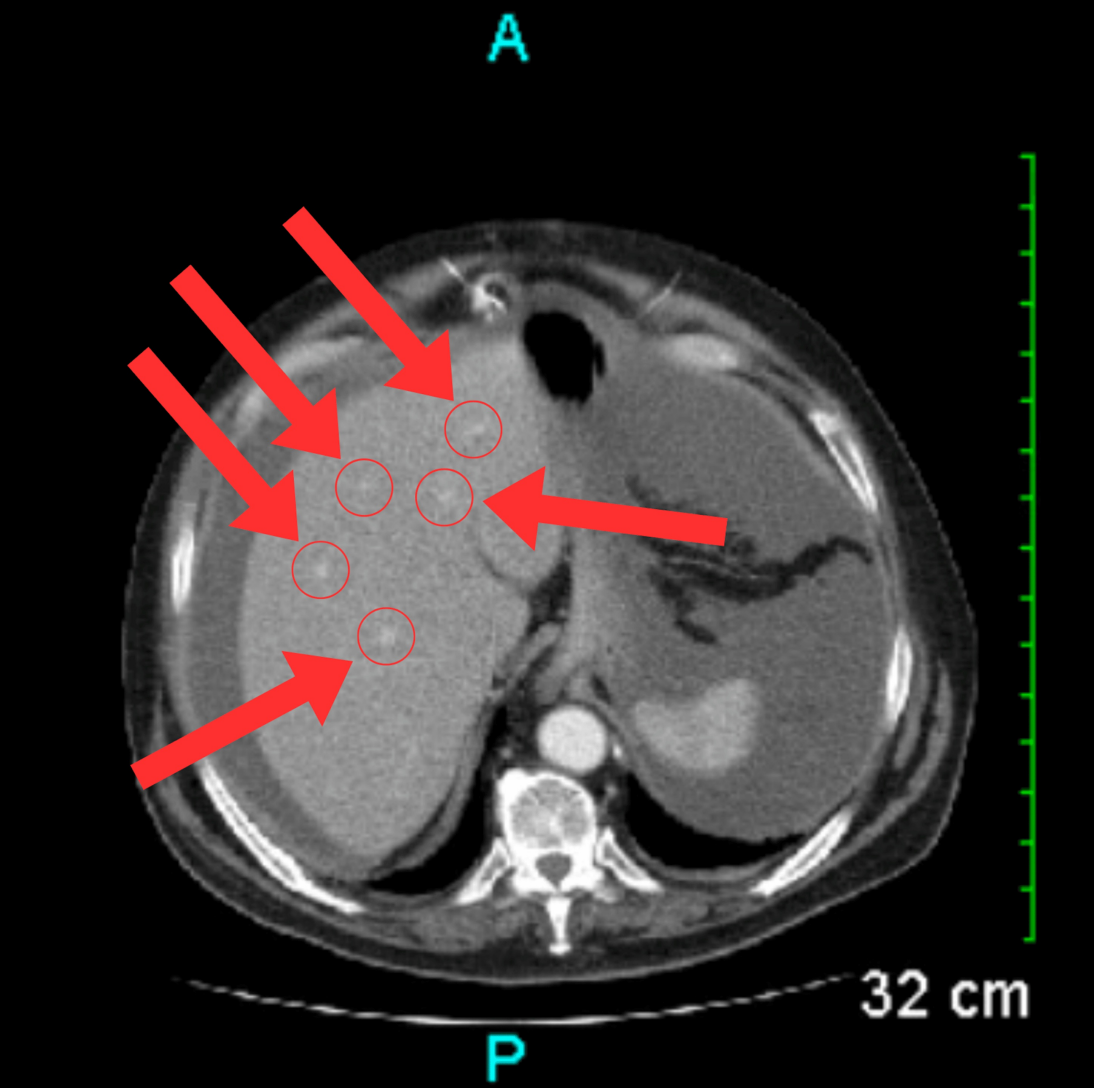
CT scan showing mild hepatic steatosis (red arrows and circles). CT: Computed tomography

**Figure 5:**
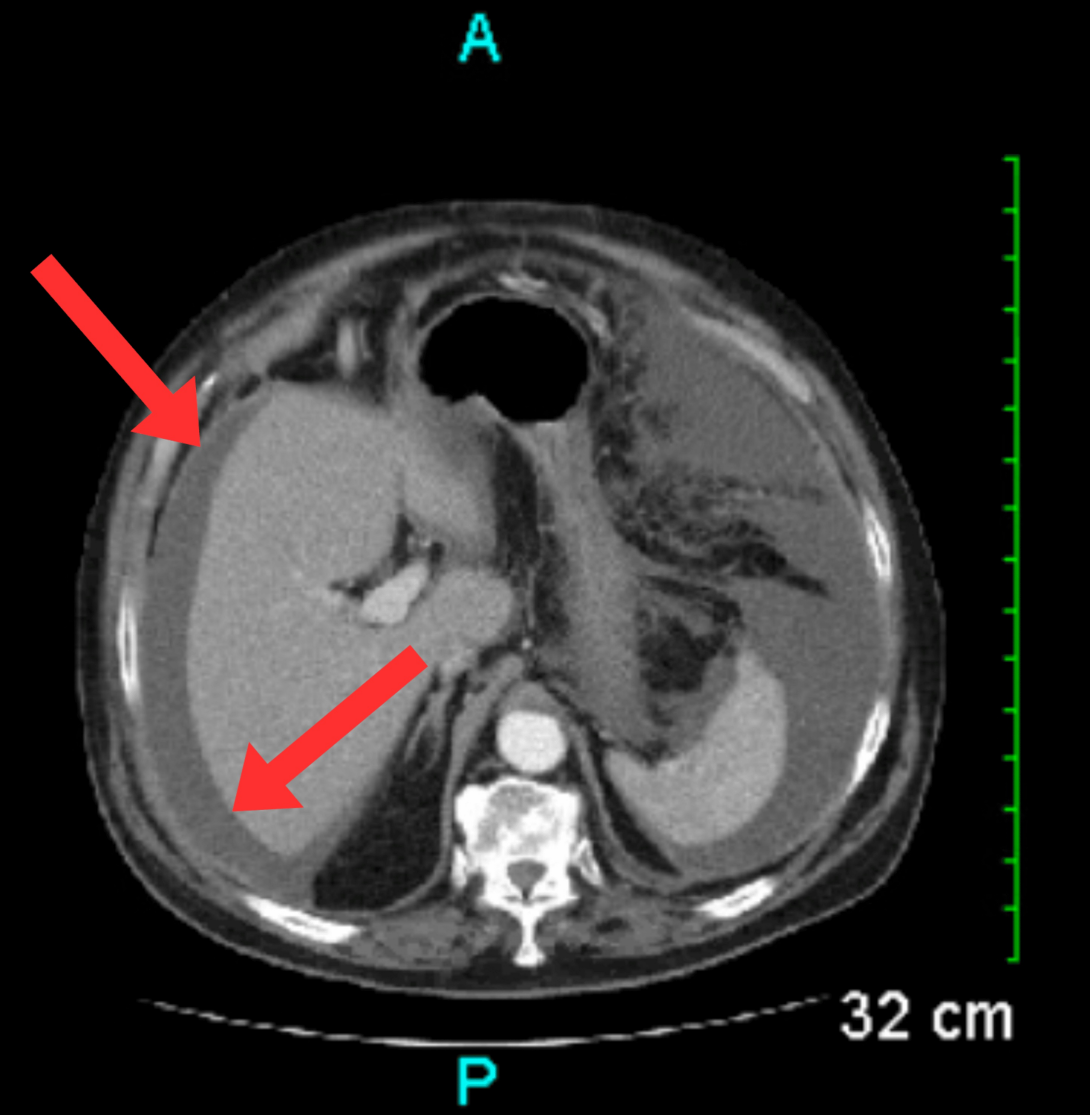
CT scan through the upper abdomen showing ascites (red arrows) adjacent to the liver. CT: Computed tomography

**Figure 6:**
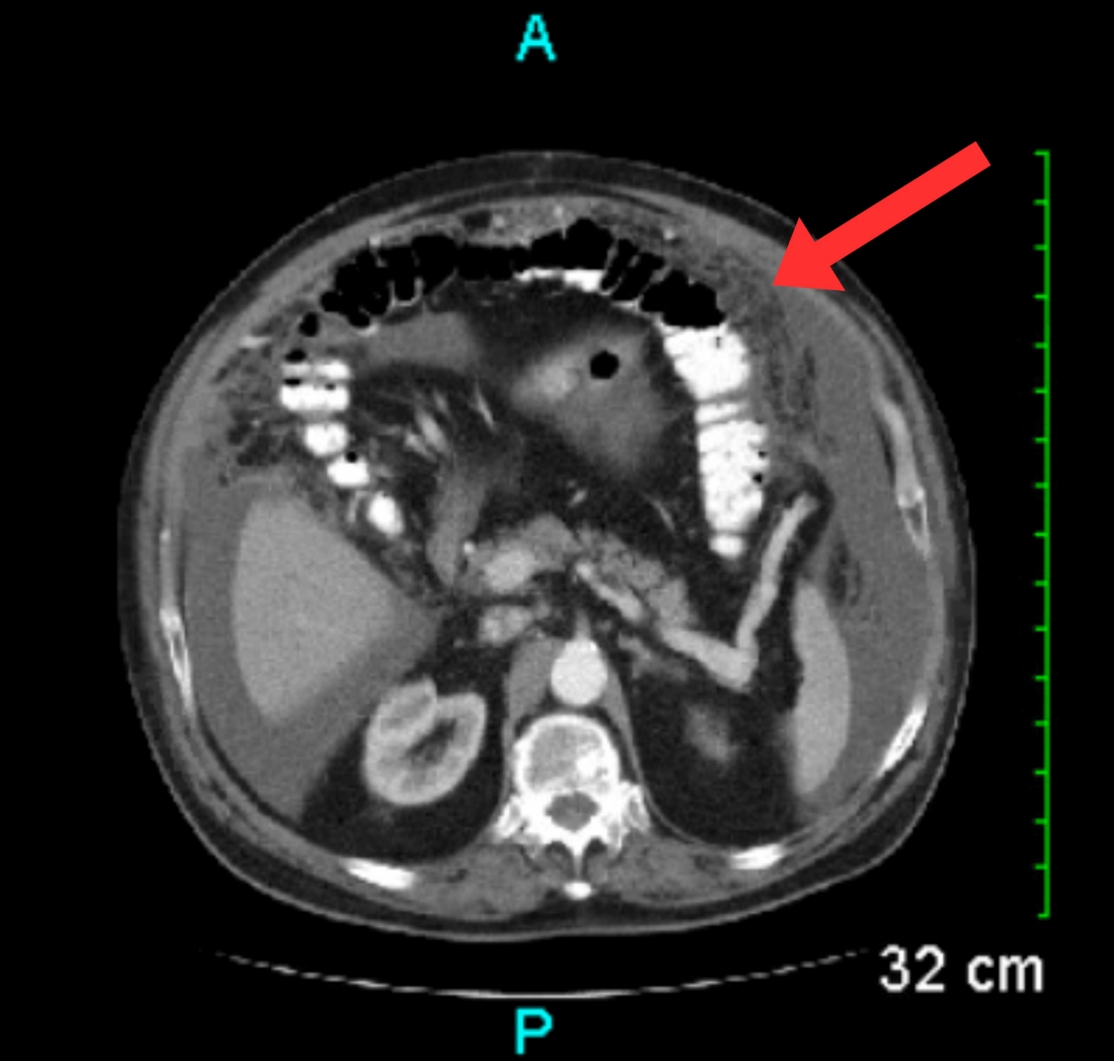
CT scan showing moderate diffuse omental edema (red arrow). CT: Computed tomography

**Figure 7:**
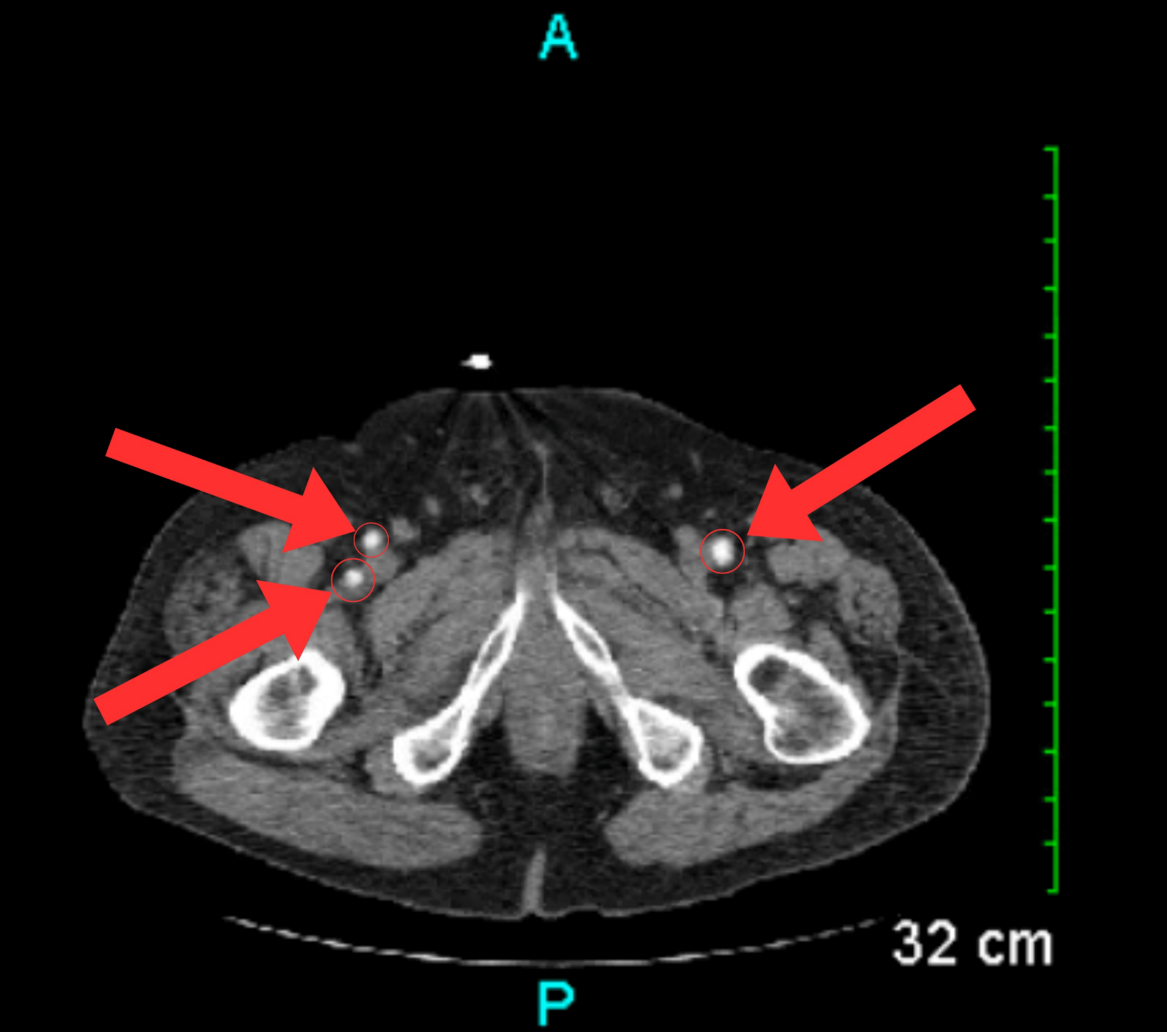
CT scan showing pelvic phleboliths (red arrows and circles). CT: Computed tomography

A chest X-ray was ordered and showed the presence of sternal wires, healed rib fractures, and reduced lung volumes (Figure 1). The laboratory workup indicated elevated serum blood urea nitrogen (BUN) levels, low serum albumin levels, and low total protein levels (Table 1). Although nonspecific, the laboratory findings may suggest underlying end-organ damage, which could potentially contribute to the development of ascites. Serial axial images were obtained through the abdomen and pelvis via CT scan with contrast. The imaging revealed several findings: a prior sternotomy (Figure 2), mild pleural calcifications (Figure 3), mild hepatic steatosis (Figure 4), diffuse peritoneal ascites (Figure 5), diffuse omental edema (Figure 6), and pelvic phleboliths (Figure 7).

**Table 1:** Initial laboratory evaluation. T_4_: Thyroxine

Test	Observed Value	Reference Range
White blood cells	9.2 × 10^3^/µL	4.0-11.0 × 10^3^/µL
Red blood cells	4.1 × 10^6^/µL	4.0-5.0 × 10^6^/µL
Hemoglobin	13.7 g/dL	12.1-15.1 g/dL
Hematocrit	47%	42%-52%
Mean corpuscular hemoglobin	28 pg/cell	27-31 pg/cell
Mean corpuscular hemoglobin concentration	35 g/dL	33-36 g/dL
Mean corpuscular volume	86 fL	80–100 fL
Platelet count	296 × 10^9^/L	150-450 × 10^9^/L
Mean platelet volume	11 fL	8-12 fL
Red cell distribution width	12%	12%-15%
Sodium	138 mmol/L	135-147 mmol/L
Potassium	4.2 mmol/L	3.5-5.0 mmol/L
Chloride	101 mmol/L	96-106 mmol/L
Carbon dioxide	28 mmol/L	23-29 mmol/L
Blood urea nitrogen	12.7 mmol/L	2.1-8.5 mmol/L
Creatinine	1.1 mg/dL	0.7-1.3 mg/dL
Glucose	81 mg/dL	70-100 mg/dL
Calcium	9.8 mg/dL	8.5-10.2 mg/dL
Albumin	3.1 g/dL	3.5-5.5 g/dL
Alkaline phosphatase	61 U/L	44-147 U/L
Alanine aminotransferase	18 U/L	7-56 U/L
Aspartate aminotransferase	29 U/L	5-40 U/L
Total bilirubin	0.4 mg/dL	0.3-1.0 mg/dL
Total protein	5.3 g/dL	6.0-8.3 g/dL
Globulin	2.7 g/dL	2.0-3.5 g/dL
Lipase	64 U/L	10-140 U/L
Magnesium	1.7 mEq/L	1.3-2.1 mEq/L
Thyroid-stimulating hormone	2.1 mU/L	0.45-4.5 mU/L
T_4_	8.9 μg/dL	5.0-12.0 μg/dL

Given these new findings, the suspicion of a primary malignancy arose, prompting the initiation of a comprehensive workup. The patient underwent esophagogastroduodenoscopy and colonoscopy, both of which were unremarkable, showing no signs of malignancy in the upper or lower gastrointestinal tracts. Additionally, tumor markers including carcinoembryonic antigen (CEA), prostate-specific antigen (PSA), cancer antigen 19-9 (CA 19-9), and alpha-fetoprotein (AFP) were measured and found to be within normal limits, further reducing the likelihood of a gastrointestinal or prostate primary malignancy.

**Figure 8:**
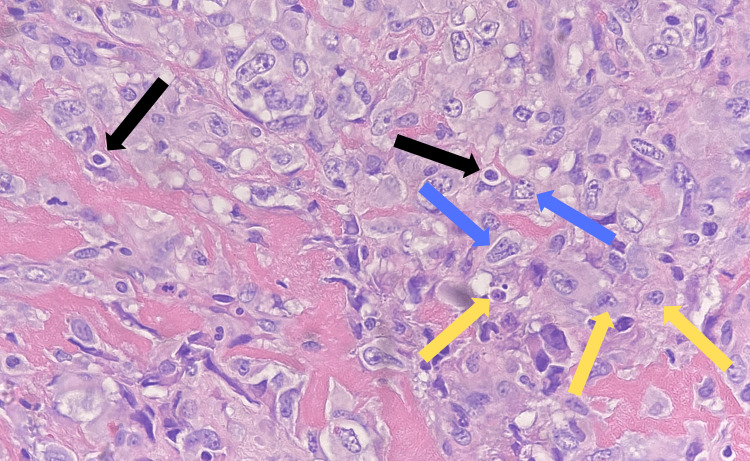
Histopathological examination showing atypical mesothelial cells with high nucleus-to-cytoplasm ratios (black arrows), prominent nucleoli (yellow arrows), and irregular nuclear membranes (blue arrows).

A therapeutic and diagnostic ultrasound-guided paracentesis was performed due to significant ascites, yielding 3.1 liters of blood-tinged fluid. A biopsy was then obtained from the peritoneal lining. Histopathological examination of the biopsy specimen revealed atypical mesothelial cells with high nucleus-to-cytoplasm ratios, prominent nucleoli, and irregular nuclear membranes, consistent with malignant pleural mesothelioma (Figure 8).

The serum-ascites albumin gradient was calculated to be <0.7, indicating that the ascites were unrelated to portal hypertension. Following the findings from the fluid analysis and CT scan of the abdomen and pelvis, the clinical decision was made to proceed with a CT-guided omental biopsy. Histopathological examination revealed tumor cells positive for p53, calretinin, WT1, and podoplanin (D2-40), confirming the diagnosis of MPM, epithelioid subtype. Considering the patient's advanced age and significant medical comorbidities, he was determined to be a poor candidate for cytoreductive surgery or chemotherapy. Given the aggressive nature and poor prognosis associated with MPM, the patient chose to decline further active treatment and opted for palliative care.

## Discussion

MPM is a rare and aggressive cancer with a generally poor prognosis. Its clinical presentation is often vague and nonspecific, necessitating a high level of suspicion, particularly in individuals with a history of cumulative asbestos exposure, especially from occupational sources. Common symptoms of MPM include abdominal distension, weight loss, and abdominal pain, all of which were observed in our patient [5]. The most frequently reported symptoms are abdominal distension and pain. Less common manifestations can include abdominal masses, new hernias, diarrhea, vomiting, and systemic symptoms such as fever and weight loss. In the early stages, patients may be asymptomatic [6]. The presence of ascites typically indicates advanced disease staging. This case highlights the critical need to consider malignant mesothelioma in the differential diagnosis when a patient presents with ascites.

When evaluating a case of ascites in the context of suspected asbestos exposure, it is important to consider a broad differential diagnosis. This should include but is not limited to, conditions such as portal hypertension, heart disease, infections, pancreatitis, nephrotic syndrome, hypoalbuminemia, and other malignancies. A thorough physical examination should assess for signs of heart failure, liver disease, and lymphadenopathy. Blood tests should be conducted to evaluate kidney and liver function. Abdominal ultrasound is the initial imaging test used to confirm the presence of ascites. If ascites are confirmed on ultrasound, abdominal paracentesis should be performed for diagnostic purposes in all patients with new-onset ascites [7]. If mesothelioma is suspected based on the analysis of ascitic fluid, a CT scan is recommended as the primary imaging modality. Although asbestos exposure is a major risk factor for mesothelioma, its association with MPM is weaker compared to its link with pleural mesothelioma [4].

The use of immunohistochemical markers is crucial for accurately diagnosing MPM. Key epithelial cell markers with high sensitivity include calretinin, WT1, and CK5/6, while connective tissue markers such as D2-40 are important in identifying mesothelial origin [4,8]. In the case of our patient, the excisional biopsy of the tumor showed extensive staining for p53, calretinin, WT1, and D2-40, while it was negative for B72.3, napsin, TF-1, CEA, and PCA. Histopathological examination revealed a proliferation of large epithelioid cells with small eosinophilic nuclei. Mesothelioma is categorized into three main types: epithelioid, sarcomatoid, and biphasic. Epithelioid mesothelioma, which is the most common and accounts for about 80% of cases, generally has a more favorable prognosis. Sarcomatoid mesothelioma is rare and is associated with a less favorable prognosis [3,9]. Biphasic mesothelioma, which exhibits features of both epithelioid and sarcomatoid types, also carries a poor prognosis similar to that of sarcomatoid mesothelioma [9].

The management of MPM primarily involves systemic chemotherapy, with the pemetrexed and cisplatin combination being the first-line treatment. This regimen has achieved a disease-stabilization rate of approximately 45%. Retrospective studies have established that cytoreductive surgery combined with hyperthermic intraperitoneal chemotherapy represents the gold standard for therapeutic intervention. Additionally, research indicates that female patients under the age of 60 generally experience better outcomes. Key prognostic factors include lymph node metastases, deep tissue invasion, and a high tumor burden, all of which are associated with a poorer prognosis and shortened survival [10]. Prognostic markers such as Ki67 and BCL2 also play a significant role in assessing MPM. Ki67, a marker of tumor proliferation, is used to evaluate tumor replication levels; higher Ki67 levels are associated with increased tumor aggression and a worse prognosis [3,11]. Conversely, BCL2 has been observed to have tumor-suppressive effects in some studies. The combination of Ki67 and BCL2 as a prognostic index has proven to be a more effective marker for predicting patient outcomes in MPM [11].

The significance of this case highlights the challenges associated with diagnosing an exceptionally rare condition. With an annual incidence in the United States estimated at just 1 in 1 million [1], diagnosing MPM requires considerable expertise. The patient's symptoms are nonspecific and can be misleading, highlighting the importance of maintaining a broad differential diagnosis and adhering to standard diagnostic protocols, especially when evaluating abdominal distension potentially caused by new-onset ascites [7]. Clinicians should be vigilant about key risk factors and well-versed in the process of achieving a definitive pathological diagnosis, including the implications of different subtypes for prognosis [3,4,9]. While current management strategies for MPM exist, it is crucial to recognize that the overall prognosis remains poor [10].

## Conclusions

MPM is a severe and aggressive cancer with a generally poor prognosis and a low median survival time. Early detection is challenging, as the disease is often asymptomatic or presents with nonspecific symptoms. A thorough patient history is crucial for raising suspicion and enabling early diagnosis. The management of MPM requires a multidisciplinary approach involving oncology, surgery, and pathology to optimize outcomes and tailor treatments to individual patient needs. Recent advances in treatment, including cytoreductive surgery combined with hyperthermic intraperitoneal chemotherapy, have shown promise in extending survival. However, due to the disease’s rarity, data remains limited, and further research is needed to explore novel therapies. Future approaches may involve cancer genomics to target specific mutations and gene markers. Given the poor prognosis, prevention remains a critical strategy to reduce mortality. The United States has taken a significant step by regulating asbestos-containing products since 1989. Ongoing research should focus on identifying additional major risk factors, as asbestos exposure may no longer be the sole significant contributor to this disease in the future.
